# Regional and sex-specific variation in BMI distribution in four sub-Saharan African countries: The H3Africa AWI-Gen study

**DOI:** 10.1080/16549716.2018.1556561

**Published:** 2019-01-17

**Authors:** Michèle Ramsay, Nigel J. Crowther, Godfred Agongo, Stuart A. Ali, Gershim Asiki, Romuald P. Boua, F. Xavier Gómez-Olivé, Kathleen Kahn, Christopher Khayeka-Wandabwa, Felistas Mashinya, Lisa Micklesfield, Freedom Mukomana, Engelbert A. Nonterah, Cassandra Soo, Hermann Sorgho, Alisha N. Wade, Ryan G. Wagner, Marianne Alberts, Scott Hazelhurst, Catherine Kyobutungi, Shane A. Norris, Abraham R. Oduro, Osman Sankoh, Halidou Tinto, Stephen Tollman, as members of AWI-Gen and the H3Africa Consortium

**Affiliations:** a Sydney Brenner Institute for Molecular Bioscience, Faculty of Health Sciences, University of the Witwatersrand, Johannesburg, South Africa; b Division of Human Genetics, National Health Laboratory Service and School of Pathology, Faculty of Health Sciences, University of the Witwatersrand, Johannesburg, South Africa; c Department of Chemical Pathology, National Health Laboratory Service, Faculty of Health Sciences, University of the Witwatersrand, Johannesburg, South Africa; d Navrongo Health Research Centre, Navrongo, Ghana; e African Population and Health Research Center, Nairobi, Kenya; f Clinical Research Unit of Nanoro, Institut de Recherche en Sciences de la Sante, Nanoro, Burkina Faso; g MRC/Wits Rural Public Health and Health Transitions Research Unit (Agincourt), School of Public Health, Faculty of Health Sciences, University of the Witwatersrand, Johannesburg, South Africa; h School of Public Health, Faculty of Health Sciences, University of the Witwatersrand, Johannesburg, South Africa; i INDEPTH Network, Accra, Ghana; j Department of Pathology and Medical Science, School of Health Care Sciences, Faculty of Health Sciences, University of Limpopo, Polokwane, South Africa; k MRC/Wits Developmental Pathways for Health Research Unit, Faculty of Health Sciences, University of the Witwatersrand, Johannesburg, South Africa; l School of Electrical & Information Engineering, University of the Witwatersrand, Johannesburg, South Africa; m Statistics Sierra Leone, Freetown, Sierra Leone; n Department of Community Medicine, College of Medicine and Allied Health Sciences, University of Sierra Leone, Freetown, Sierra Leone

**Keywords:** BMI, SSA, regional variation, sex-specific variation, obesity, CMD

## Abstract

**Background**: African populations are characterised by diversity at many levels including: demographic history, genetic ancestry, language, wealth, socio-political landscape, culture and behaviour. Several of these have a profound impact on body fat mass. Obesity, a key risk factor for cardiovascular and metabolic diseases, in the wake of the epidemiological and health transitions across the continent, requires detailed analysis together with other major risk factors.

**Objective**: To compare regional and sex-specific body mass index (BMI) distributions, using a cross-sectional study design, in adults aged 40–60 years across six study sites in four sub-Saharan African (SSA) countries and to compare the determinants of BMI at each.

**Methods**: Anthropometric measurements were standardised across sites and BMI calculated. Median BMI and prevalence of underweight, lean, overweight and obesity were compared between the sexes and across sites. Data from multivariable linear regression models for the principal determinants of BMI were summarised from the site-specific studies.

**Results**: BMI was calculated in 10,702 participants (55% female) and was significantly higher in women than men at nearly all sites. The highest prevalence of obesity was observed at the three South African sites (42.3–66.6% in women and 2.81–17.5% in men) and the lowest in West Africa (1.25–4.22% in women and 1.19–2.20% in men). Across sites, higher socio-economic status and educational level were associated with higher BMI. Being married and increased dietary intake were associated with higher BMI in some communities, whilst smoking and alcohol intake were associated with lower BMI, as was HIV infection in the regions where it was prevalent.

**Conclusion**: In SSA there is a marked variation in the prevalence of obesity both regionally and between men and women. Our data suggest that the drive for social upliftment within Africa will be associated with rising levels of obesity, which will require the initiation of targeted sex-specific intervention programmes across specific African communities.

## Background

The global increase in obesity is accelerating [1–3] and in 2015 high BMI accounted for 4 million deaths, with over-two thirds due to cardiovascular disease. In Africa, the rapid rise in obesity in countries undergoing the nutrition transition [4] is of great concern as it is accompanied by an increase in the prevalence of associated cardiometabolic diseases and premature deaths. Obesity trends in Africa (1980 to 2014), modelled across 1.2 million African participants, showed that the age-standardised mean BMI (kg/m^2^) increased from 21.0 to 23.0 in men and 21.9 to 24.9 in women, with significant regional and sex differences [2]. Increases in southern and northern regions of Africa were above the global estimates, whereas those in West and Central Africa were below [1,2].

The small number of studies on the epidemiology of obesity in Africa, often with modest sample sizes, do not adequately reflect the extensive biological and sociodemographic heterogeneity of the continent. It is important to explore and understand these country-specific differences as they may influence our understanding of obesity and its consequences across the continent. In the Human Heredity and Health in African (H3Africa) Consortium AWI-Gen Study (Africa Wits-INDEPTH partnership for Genomic studies) we have collected data from 10,702 participants between the ages of 40 and 60 years, from six study sites in four African countries [5,6]. In this special issue of Global Health Action, each of the six AWI-Gen participant Centres has reported individually on the distribution of BMI and its determinants [7–12]. They have analysed BMI in the context of participant socio-demographic factors (sex, age, ethnicity, educational status, employment, household assets, partner/marital status), behaviour (smoking/tobacco exposure, alcohol consumption and exercise) and other environmental factors (infections and pesticide exposure), and used an hierarchical modelling approach, and in one case multiple linear regression, to examine their association with BMI. Data collection was standardised across the study sites and are described in detail in Ail et al. [5], published in this same special issue.

The main aim of the current paper was to synthesize the key outcomes and conclusions from the individual papers, to examine the regional and sex-specific differences in BMI and the prevalence of underweight, leanness, overweight and obesity. A further aim was to compare the key factors influencing BMI across the six study sites.

## Methods

### Participant recruitment and measurement of variables that modulate body fat mass

Methods used for the recruitment of study participants are described in detail in the individual papers and in [5]. These papers also describe the methods used for the assessment of variables that may influence BMI. These variables include age, sex, ethnicity, socio-economic status, household crowding, education level, marital status, physical activity, sedentary time, night time sleep duration, smoking, use of smokeless tobacco, dietary intake, alcohol intake, pesticide use, HIV and TB status, parity and menopausal stage. The main conclusions are summarised and compared across study sites.

### BMI and categories of obesity

Body mass index (BMI) calculated as kg/m^2^, was analysed as both a continuous variable and categorized according to the standards adopted by the World Health Organization [13], using the following cut-offs: underweight (BMI<18.5 kg/m^2^), lean (BMI ≥18.5 and <25kg/m^2^), overweight (BMI≥25 and <30 kg/m^2^) and obese (BMI≥30 kg/m^2^).

### Statistical analyses

The BMIs were not normally distributed and were therefore expressed as medians with interquartile ranges. Sex comparisons for BMI at each study site were assessed using the Mann-Whitney U test. The Kruskal-Wallis test was used to compare BMI across study sites, stratified by sex. Differences between men and women for the prevalence of the four different BMI categories were assessed using χ^2^ 2 × 4 contingency tables.

## Results

**Figure 1.**
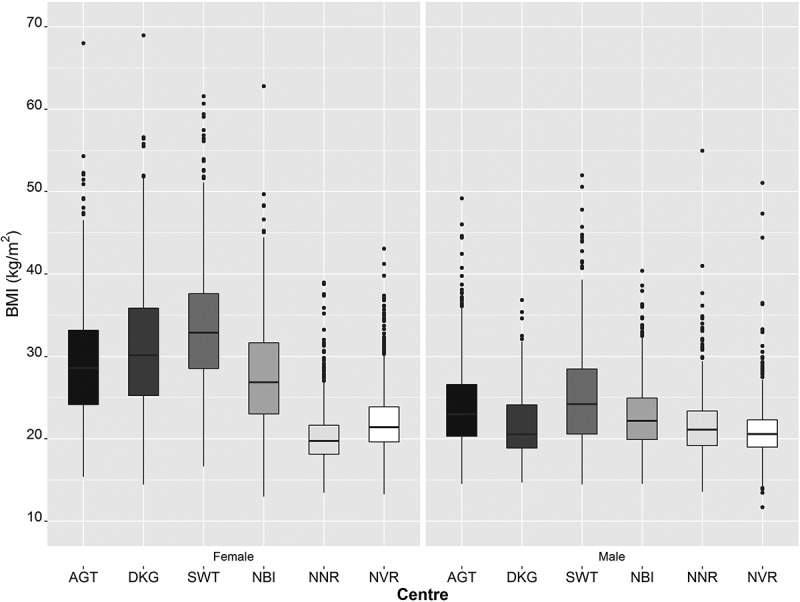
Median BMI (kg/m^2^) values (box and whisker plots showing the median, interquartile ranges and outliers) across the six AWI-Gen study sites stratified by sex. From left to right are the Centres from South Africa (Agincourt (AGT); Dikgale (DKG); and Soweto (SWT)), East Africa (Nairobi in Kenya (NBI)); and West Africa (Nanoro, Burkina Faso (NNR) and Navrongo, Ghana (NVR)).

The BMI distribution for men and women at each of the six study sites is shown in the box-and-whisker plots in Figure 1 and as a comparison of the median BMI levels in Table 1. There was a highly significant difference in BMI between men and women (p < 0.0001) at all sites with women being more obese than men, except at Nanoro (Burkina Faso) where women had a lower BMI. These data show that there is greater variation in BMI across study sites for women, with the two West African study sites having the lowest BMI measures.

**Table 1.** Comparison of BMI measurements by sex in the six AWI-Gen Centres (40–60 years of age).

			N			BMI	
Centre	Country	Urbanicity	Total	Women	Men	Women	Men
Soweto	South Africa	Urban	2030	1005	1025	32.9 [28.5, 37.6]	24.2 [20.6, 28.5]***
Agincourt	South Africa	Semi-rural	1465	573	892	28.6 [24.2, 33.2]	23.0 [20.3, 26.6]***
Dikgale	South Africa	Semi-rural	1167	356	811	30.1 [25.2, 35.9]	20.6 [18.9, 24.1]***
Nairobi	Kenya	Urban	1942	886	1056	26.9 [23.0, 31.7]	22.1 [19.9, 24.9]***
Nanoro	Burkina Faso	Rural	2084	1045	1039	19.7 [18.1, 21.6]	21.1 [19.2, 23.4]***
Navrongo	Ghana	Rural	2014	923	1091	21.4 [19.6, 23.9]	20.6 [19.0, 22.3]***

Data given as median [interquartile range]; Women vs. Men ***p < 0.0001 (Mann-Whitney U test).

Pairwise comparisons between all sites, stratified by sex, show significant differences for all comparisons in women except for Dikgale versus Agincourt (both in South Africa), where no significant difference was observed (p = 0.095). For men, significant differences were noted for all comparisons with the exception of Dikgale where men have similar BMI to those in West Africa (Nanoro and Navrongo) (Supplementary Tables S1A and S1B).

**Figure 2.**
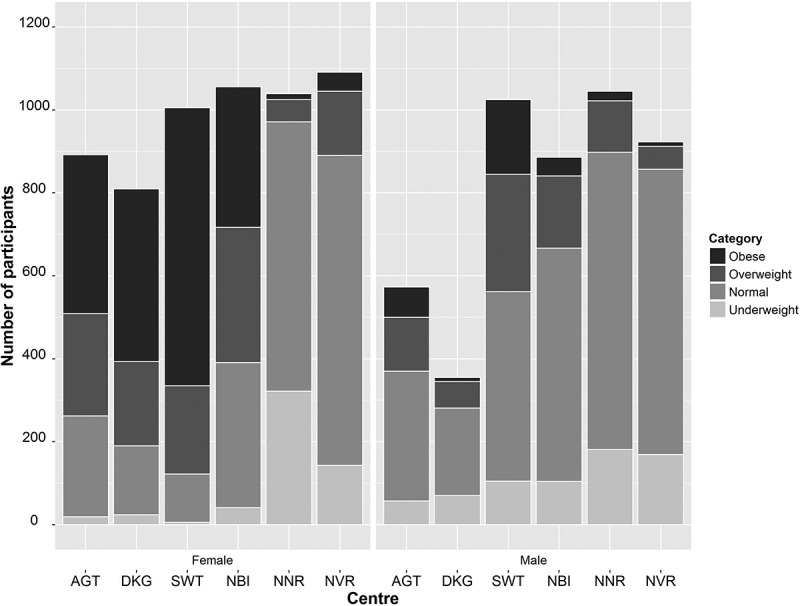
Distribution of obesity categories (obese, overweight, lean (normal) and underweight) across the six AWI-Gen data collection sites, stratified by sex. From left to right are the centres from South Africa (Agincourt (AGT); Dikgale (DKG); and Soweto (SWT)), East Africa (Nairobi in Kenya (NBI)); and West Africa (Nanoro, Burkina Faso (NNR) and Navrongo, Ghana (NVR)). The bars represent the number of individuals recruited at each Centre.

The BMI data were divided into four categories, underweight, lean, overweight and obese, and the numbers of participants in each category, by site and sex, are shown in Figure 2. Table 2 shows the prevalence of the four BMI categories at each study site and compares them between the sexes. At five study sites the women have a higher proportion of obese individuals than men whereas men have a higher proportion of both underweight and lean individuals compared to women. The exception is Nanoro, where although obesity is rare, there is a tendency for men to have a higher prevalence of overweight and obesity than women, but a lower prevalence of underweight. These data clearly show the much higher prevalence of overweight and obesity in women in South Africa and Kenya when compared to the West African study sites and a higher level of underweight and leanness in the latter, especially in women.

**Table 2.** Comparison of the prevalence of BMI categories between men and women at each AWI-Gen Centre.

Centre	Sex	Underweight (%)	Lean (%)	Overweight (%)	Obese (%)	P-value*
Soweto	Women	0.6	11.5	21.2	66.6	<0.0001
	Men	10.2	44.6	27.6	17.5	
Agincourt	Women	2.1	27.5	28.0	42.3	<0.0001
	Men	10.0	55.2	22.9	11.8	
Dikgale	Women	3.0	20.5	25.1	51.4	<0.0001
	Men	19.7	59.5	18.0	2.8	
Nairobi	Women	3.9	33.1	30.9	32.1	<0.0001
	Men	11.7	63.5	19.6	5.1	
Nanoro	Women	31.0	62.5	5.2	1.3	<0.0001
	Men	17.3	68.6	11.9	2.2	
Navrongo	Women	13.1	68.5	14.2	4.2	<0.0001
	Men	18.3	74.5	6.0	1.2	

*p-values from a χ^2^ 2 × 4 contingency table.

The information in Table 3 shows the variables associated with BMI in men and women across all six study sites. One or more of each of the sociodemographic factors (SES, education or marital status) were significantly associated with BMI for each sex at each study site, with the exception of women in Nairobi. Smoking was negatively associated with BMI in men and/or women at each of the six sites. Alcohol consumption was associated with BMI in men or women at each of five study sites with the exception being Soweto (South Africa). In Nairobi the association between alcohol consumption and BMI was positive, but at the other sites it was negative. Dietary intake (either sugar-sweetened beverages, fruit and vegetable or bread consumption) was positively associated with BMI in either men or women at three sites. Infection with HIV was negatively associated with BMI at all three South African study sites and in Nairobi. At three sites (Soweto, Nairobi and Navrongo), ethnicity was significantly associated with BMI. Sleep duration was negatively associated with BMI in both sexes at Navrongo, and in men at Dikgale.

**Table 3.** Sex-stratified hierarchical models showing demographic, socio-economic, behavioural and biological factors associated with BMI across six African study sites.

Centre (Ref.)	Men		Women	
	R^2^	Significant associations	R^2^	Significant associations
Soweto [10]	0.26	Marital status, SES, Smoking (-), Physical activity (-), HIV (-)	0.14	Tswana Ethnicity (-), Education (-), Smoking (-), HIV status (-)
Agincourt [12]		Marital status, SES, Smoking (-)		Marital status, Education, Smoking (-), Alcohol (-), Sugar-sweetened beverages, HIV (-)
Dikgale [9]	0.25	Marital status, SES, Smoking (-), Alcohol (-), HIV (-)	0.12	Age (-), SES, Smoking (-), Sleep duration (-), HIV (-)
Nairobi [7]	0.20	SES, Smoking (-), Bread, HIV (-), TB (-)	0.19	Smoking (-), Alcohol, Kikuyu ethnicity, HIV (-), TB (-)
Nanoro [8]	0.26	Age (-), Marital status, Education, SES, Alcohol (-), Smoking (-), Fruit and vegetables	0.16	Education, Smokeless tobacco (-), Bread
Navrongo [11]	0.20	Age (-), Nankana ethnicity (-), Education, SES, Smoking (-), Alcohol (-), Sedentary time, Sleep duration (-), Pesticide use	0.19	Age (-), Nankana ethnicity (-), Education, SES, Smokeless tobacco (-), Sleep duration (-)

Directionality of associations is positive unless stated otherwise. Marital status: Being married or co-habiting is associated with higher BMI. Details for each study site are provided in the Special Issue [7–12].

## Discussion

Obesity is one of the six risk factors identified as targets for reducing non-communicable diseases (NCDs) by 25% by the year 2025 [14–16]. It is predicted that the probability of dying from one of the four main NCDs, including cardiovascular diseases, diabetes, cancer and obstructive lung disease, will increase between the ages of 30 and 70 years in Africa, while decreasing in the rest of the world [15].

In this study, we report and compare BMI distribution and WHO classifications for underweight, lean, overweight and obesity between the six AWI-Gen Centres. In the accompanying set of papers, including one from each Centre, the risk factors and correlates with obesity were examined [7–12]. At five of the six study sites women had significantly higher BMI compared to men, whereas in Nanoro, men had higher BMI. Obesity was more common in South Africa with intermediate prevalence in East Africa and the lowest prevalence in West Africa. The low prevalence of obesity in the West African sites may be due to socio-economic effects, since a majority of these populations are reliant on subsistence farming.

The most consistent correlates with higher BMI were SES and education level (Table 3). SES was measured according to household assets and then grouped into quintiles within each site, ranking individuals from the least poor to the poorest. The trend of higher BMI with greater wealth is a characteristic of low- and middle-income countries whereas the reverse is true in most high-income countries [17].

Marital status had a significant association with BMI at many of the study sites, particularly in men, with married participants having higher BMI than those who were not married. This was observed in men in Soweto, Agincourt, Dikgale and Nanoro, and in women from Agincourt.

Smoking and alcohol consumption were associated with lower BMI, but this was not significant in some of the communities, or applied only to one sex. In West Africa it is extremely rare for women to smoke, and in the other regions they are less likely to smoke than men. In Nanoro and Navrongo women who chew tobacco were shown to have a lower BMI. At a number of the study sites (Dikgale, Nanoro and Navrongo) alcohol consumption was significantly associated with lower BMI in men, and in women (Agincourt), but in Nairobi it was associated with higher BMI in women who reported non-problematic alcohol intake. The sites where alcohol was associated with lower BMI, reported that this association was seen in participants with problematic alcohol intake which is supported by data from other studies [18,19]. Interestingly self-reported ethnicity, as a proxy for genetic ancestry, was associated with BMI in Soweto, Nairobi and Navrongo, suggesting that background genetic variation and gene environment interactions likely make a substantive contribution to BMI. These associations will need to be explored in more detail together with an improved understanding of the genetic contributions to the trait.

Most of the study sites (with the exception of men in Soweto) showed no correlation of BMI with the amount of moderate and vigorous physical activity, although it is well recognised that increased physical activity has significant health benefits.

At the four study sites in South and East Africa where HIV infection is common (14–34% in South Africa and 12% in Nairobi, in the AWI-Gen 40–60 year cohort – data not shown), there was a significant inverse association between infection status and BMI. This needs to be explored more fully taking into consideration the duration of infection and being on anti-retroviral treatment. Tuberculosis infection was also associated with lower BMI in both sexes in Nairobi. Only one site, Navrongo, reported on pesticide exposure and showed a positive correlation with BMI, warranting further investigation.

Sex as a biological variable in obesity has been studied extensively and its effects on BMI can be attributed to biological pathways and to behavioural patterns that are influenced by cultural practices and socioeconomic status [20]. Much has been written about biological differences between men and women, including body composition and differences in body fat distribution, as well as the influence of hormone levels and percentage fat (absolute and relative proportions of yellow fat). However, dietary patterns and preferences, as well as access to food, have socioeconomic and cultural influences on obesity. In low- and middle-income countries women tend to be obese more often than men, whereas this is not the case in wealthy nations. The high levels of obesity in South Africa and rising levels in Kenya are having have a profound effect on health.

Among African men, reducing raised blood pressure and smoking are the most important risk factors for premature NCD-related deaths, with obesity being fifth out of seven risk factors. Among African women, however, raised blood pressure and obesity are the top two risk factors for premature NCD-related deaths (Kontis et al. 2015). Adiposity is the most important modifiable risk factor for diabetes, and together with raised blood pressure, cholesterol and glucose, is leading to an increase in cardiovascular diseases [21–25].

Although BMI is the most commonly used measure of total body fat mass, it is recognised that it may not be the best measure of adiposity for assessing cardiometabolic disease (CMD) risk. Central obesity, including waist circumference and visceral fat, may be better predictors and will be explored further in future AWI-Gen studies. Several of the AWI-Gen Centres reported BMI association with ethnicity, suggesting important genetic contributions to obesity. A series of genome-wide and candidate gene association studies, as well as gene-environment interaction studies, will be performed using the H3Africa SNP genotyping array. Using our extensive data, AWI-Gen provides an opportunity to explore environmental and genetic factors associated with CMD across different African settings.

The AWI-Gen study has highlighted regional and sex differences in BMI distribution in middle-aged adults across four African countries. The strengths of the study include harmonized data collection, a large sample size, and the measurement of many potential confounders and modifiers of body fat mass. Phase 1 of the AWI-Gen study has generated base-line data for the study communities and Phase 2 will collect a second wave of data after a ~ five-year interval, developing cohorts where the change in CMD outcomes and risk factors can be explored. The AWI-Gen Phase 1 data are from participants aged 40 to 60 years and therefore conclusions are restricted to this age group, and although there was a spread of rural, semi-rural and urban study sites, we did not represent them in each country or region. Furthermore, several behavioural traits were only partially explored and relied on self-reported information that could be biased, especially in the context of communities that have been followed over extended periods as part of an HDSS. There is little detail on dietary intake and the associations should be interpreted with caution. The generalizability of the study outcomes is a limitation, but the study has value in its careful documentation and in its potential to inform regional public health approaches and priorities towards lowering cardiometabolic disease risk factors, such as obesity.

## Paper context

AWI-Gen aims to explore genetic and environmental contributions to body composition and cardiometabolic diseases and risk factors in Africans. We recruited 10,702 adults (40 to 60 years) from six study sites across four sub-Saharan African countries. Obesity is common in South and East Africa and rare in West Africa, with significant sex differences. BMI distribution is the combined result of SES, lifestyle, culture, sex and underlying biological factors and genetic variation, with considerable variation across Africa.
