# Supplementary material for: Regional and sex-specific variation in BMI distribution in four sub-Saharan African countries: The H3Africa AWI-Gen study
Source: Glob Health Action. 2019 Jan 17;11(Suppl 2):1556561. doi: 10.1080/16549716.2018.1556561 (PMC6407581; doi:10.1080/16549716.2018.1556561)
Supplement: Supplemental Material [file ZGHA_A_1556561_SM8288.docx]

**Supplementary Tables S1A & S1B**

**Comparison of BMI across sites for women (Table S1A) and men (Table S1B)**

**Table S1A. BMI comparisons between study sites for women**

| **Centre**  **(median BMI)** | **Sites** | | | | | |
| --- | --- | --- | --- | --- | --- | --- |
|  | **Soweto** | **Agincourt** | **Dikgale** | **Nairobi** | **Nanoro** | **Navrongo** |
| ***Soweto (32.9)*** |  | <0.0001 | <0.0001 | <0.0001 | <0.0001 | <0.0001 |
| ***Agincourt (28.6)*** | <0.0001 |  | 0.0953 | 0.0002 | <0.0001 | <0.0001 |
| ***Dikgale (30.1)*** | <0.0001 | 0.0953 |  | <0.0001 | <0.0001 | <0.0001 |
| ***Nairobi (26.9)*** | <0.0001 | 0.0002 | <0.0001 |  | <0.0001 | <0.0001 |
| ***Nanoro (19.7)*** | <0.0001 | <0.0001 | <0.0001 | <0.0001 |  | <0.0001 |
| ***Navrongo (21.4)*** | <0.0001 | <0.0001 | <0.0001 | <0.0001 | <0.0001 |  |

Data given as p-values (Kruskal-Wallis test)

**Table S1B. BMI comparisons between study sites for men**

| **Centre**  **(median BMI)** | **Sites** | | | | | |
| --- | --- | --- | --- | --- | --- | --- |
|  | **Soweto** | **Agincourt** | **Dikgale** | **Nairobi** | **Nanoro** | **Navrongo** |
| ***Soweto (24.2)*** |  | 0.0235 | <0.0001 | <0.0001 | <0.0001 | <0.0001 |
| ***Agincourt (23.0)*** | 0.0235 |  | <0.0001 | 0.0184 | <0.0001 | <0.0001 |
| ***Dikgale (20.6)*** | <0.0001 | <0.0001 |  | <0.0001 | 1.0000 | 0.2757 |
| ***Nairobi (22.1)*** | <0.0001 | 0.0184 | <0.0001 |  | <0.0001 | <0.0001 |
| ***Nanoro (21.1)*** | <0.0001 | <0.0001 | 1.0000 | <0.0001 |  | 0.0007 |
| ***Navrongo (20.6)*** | <0.0001 | <0.0001 | 0.2757 | <0.0001 | 0.0007 |  |

Data given as p-values (Kruskal-Wallis test)
